# Supplementary material for: The Cas10 nuclease activity relieves host dormancy to facilitate spacer acquisition and retention during type III-A CRISPR immunity
Source: bioRxiv. 2024 Feb 12:2024.02.11.579731. Preprint. [Version 1] doi: 10.1101/2024.02.11.579731 (PMC10888962; doi:10.1101/2024.02.11.579731)
Supplement: 1 — Figure S1. Targeting of the ΦNM4γ4 phage by the S. epidermidis type III-A CRISPR-Cas system. (A) Genome of the ΦNM4γ4 phage. Arrows indicate the two promoter, PE and PL, that drive transcription of early- and late-expressed genes. The location of the targets of the different spacers used in this study is shown. The site of insertion of gfp to generate ΦNM4γ4gfp is also indicated. (B) S. epidermidis type III-A CRISPR-cas locus. White boxes, repeats; colored boxes, spacers. Mutations introduced into cas10 and csm6 in this study are shown. (C) Spacer frequency, calculated as the fraction of spacer reads, for the library of spacers cloned into pCRISPR. Spacer sequences matching the plus and minus strands of the ΦNM4γ4 genome are plotted separately. (D) Enrichment ratio of spacers targeting the plus or minus strands of the ΦNM4γ4 DNA 24 hours after phage infection of staphylococci carrying pCRISPR, plotted according to their genomic position. (E) Abundance (in normalized reads) of ΦNM4γ4 RNA-seq reads, obtained at 5, 15 or 30 minutes after infection, mapped to the viral genome. (F) Correlation of target transcript expression (5 minutes after infection; shown as normalized RNA-seq reads) and enrichment of the corresponding targeting spacer (5 hours after infection). Pearson r coefficient is shown. (G) Time-course fluorescence microscopy of staphylococci at 0, 60 and 360 minutes after infection with ΦNM4γ4GFP or uninfected. Figure S2. Targeting of the ΦNM4γ4 phage by a S. epidermidis type III-A CRISPR-Cas system carrying the dcsm6 allele. (A) Spacer frequency, calculated as the fraction of spacer reads, for the library of spacers cloned into pCRISPR(dcsm6). Spacer sequences matching the plus and minus strands of the ΦNM4γ4 genome are plotted separately. (B) Enrichment ratio of spacers targeting the plus or minus strands of the ΦNM4γ4 DNA 24 hours after phage infection of staphylococci carrying pCRISPR(dcsm6), plotted according to their genomic position. (C) Number of uniqu [file NIHPP2024.02.11.579731V1-supplement-1.pdf]

bioRxiv preprint doi: https://doi.org/10.1101/2024.02.11.579731; this version posted February 12, 2024. The copyright holder for this preprint (which was not certified by peer review) is the author/funder, who has granted bioRxiv a license to display the preprint in perpetuity. It is made available under aCC-BY 4.0 International license.

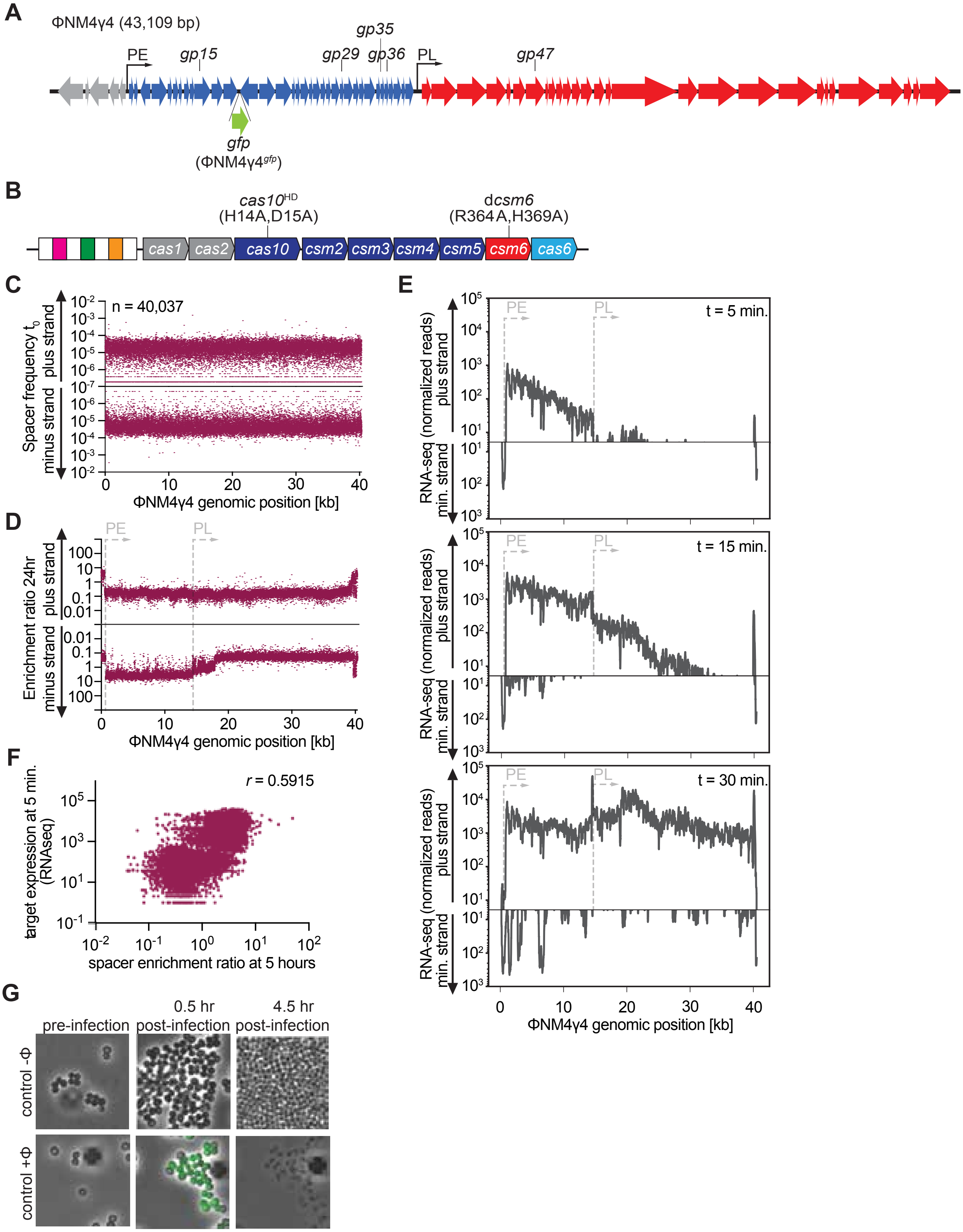

Supplementary Figure 1. Aviram, Shilton *et al.*

bioRxiv preprint doi: https://doi.org/10.1101/2024.02.11.579731; this version posted February 12, 2024. The copyright holder for this preprint (which was not certified by peer review) is the author/funder, who has granted bioRxiv a license to display the preprint in perpetuity. It is made available under aCC-BY 4.0 International license.

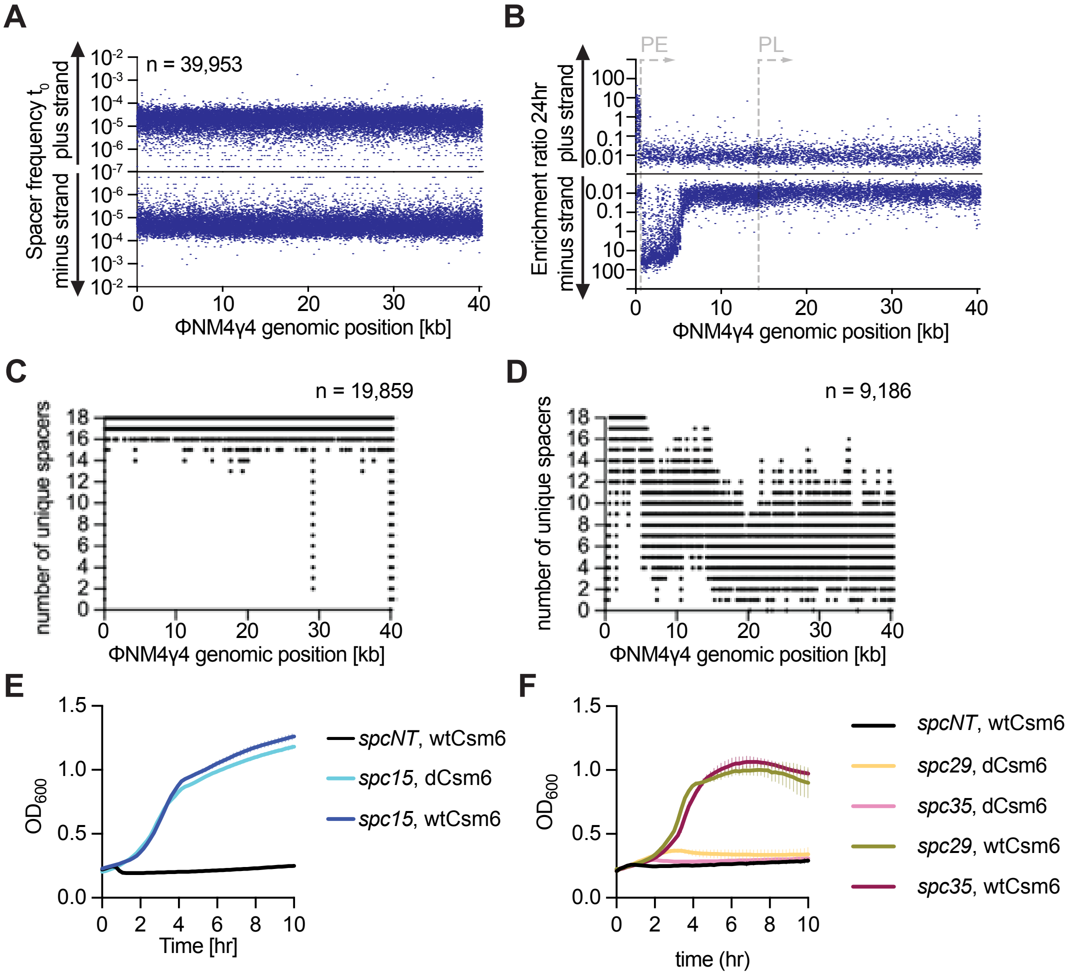

Supplementary Figure 2. Aviram, Shilton *et al*.

bioRxiv preprint doi: https://doi.org/10.1101/2024.02.11.579731; this version posted February 12, 2024. The copyright holder for this preprint (which was not certified by peer review) is the author/funder, who has granted bioRxiv a license to display the preprint in perpetuity. It is made available under aCC-BY 4.0 International license.

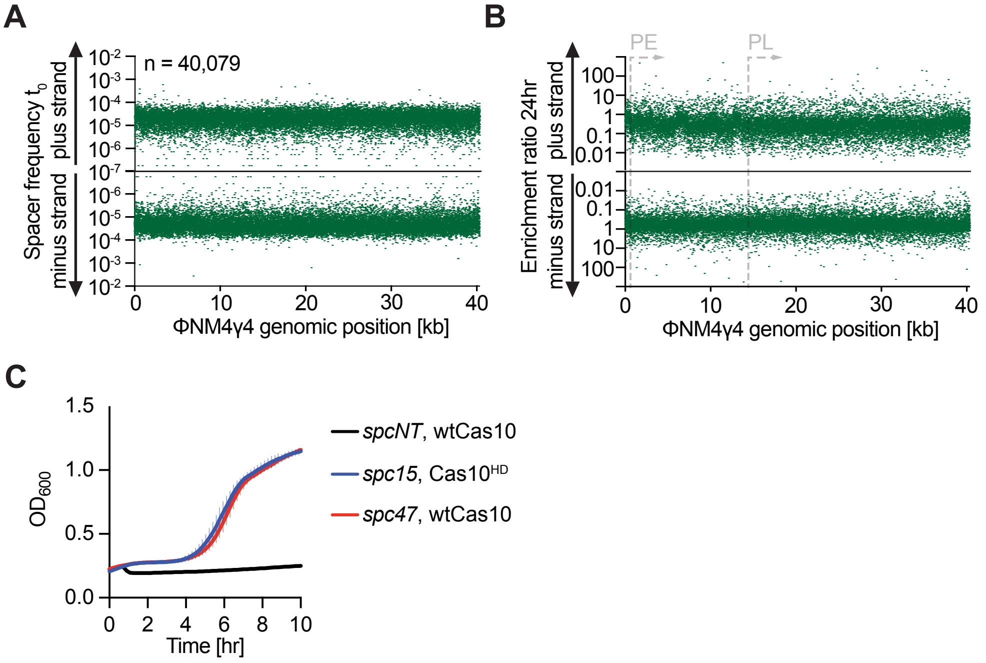

Supplementary Figure 3. Aviram, Shilton *et al*.

bioRxiv preprint doi: https://doi.org/10.1101/2024.02.11.579731; this version posted February 12, 2024. The copyright holder for this preprint (which was not certified by peer review) is the author/funder, who has granted bioRxiv a license to display the preprint in perpetuity. It is made available under aCC-BY 4.0 International license.

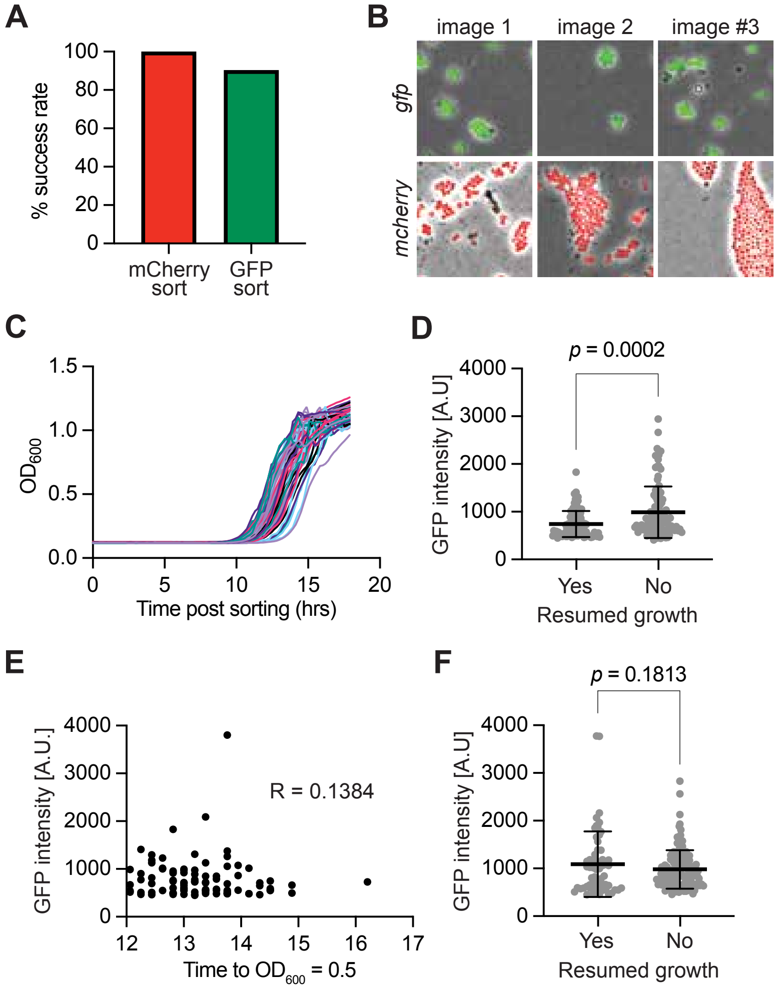

Supplementary Figure 4. Aviram, Shilton *et al*.

bioRxiv preprint doi: https://doi.org/10.1101/2024.02.11.579731; this version posted February 12, 2024. The copyright holder for this preprint (which was not certified by peer review) is the author/funder, who has granted bioRxiv a license to display the preprint in perpetuity. It is made available under aCC-BY 4.0 International license.

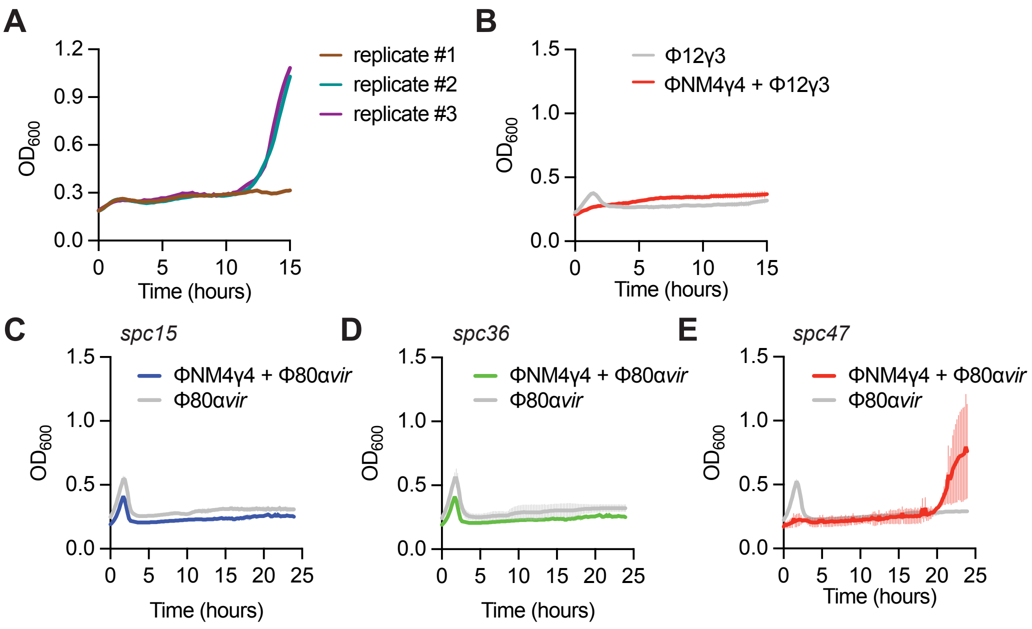

Supplementary Figure 5. Aviram, Shilton *et al*.
